# Assessing the use of an essential health package in a sector wide approach in Malawi

**DOI:** 10.1186/1478-4505-9-4

**Published:** 2011-01-17

**Authors:** Cameron Bowie, Takondwa Mwase

**Affiliations:** 1 Department of Community Health, College of Medicine, University of Malawi, Blantyre, 3, Malawi; 2 Abt Associates Inc, PO Box 30846, Lilongwe, Malawi

## Abstract

**Background:**

The sector wide approach (SWAp) used in many developing countries is difficult to assess. One way is to consider the essential health package (EHP) which is commonly the vehicle for a SWAp's policies and plans. It is not possible to measure the impact of an EHP by measuring health outcomes in countries such as Malawi. But it is possible to assess the choice of interventions and their delivery in terms of coverage. This paper describes an attempt to assess the Malawi SWAp through its EHP using these available measures of technical efficiency.

**Methods:**

A burden of disease model was used to identify the priority diseases and their estimated incidence. Data from the health management information system (HMIS) were used to measure the coverage of these interventions. A review of the cost-effectiveness of the chosen and potential interventions was undertaken to assess the appropriateness of each intervention used in the EHP. Expenditure data were used to assess the level of funding of the EHP.

**Results:**

33 of the 55 EHP interventions were found to be potentially cost-effective (<$150/DALY), 12 were not so cost-effective (>$150/DALY) and cost-effective estimates were not available for ten. 15 potential interventions, which were cost-effective and tackling one of the top 20 ranked diseases, were identified.

Provision had increased in nearly all EHP services over the period of the SWAp. The rates of out patient attendances and inpatient days per 1000 population had both increased from 929 attendances in 2002/3 to 1135 in 2007/08 and from 124 inpatient days in 2002/03 to 179 in 2007/08.

However, by 2007/08 the mean gap between what was required and what was provided was 0.68 of the estimated need. Two services involving the treatment of malaria were overprovided, but the majority were underprovided, with some such as maternity care providing less than half of what was required.

The EHP was under-funded throughout the period covering on average 57% of necessary costs. By 2007/08 the funding paid by SWAp partners including the government of Malawi to fund the EHP was at US$13.5 per capita per annum, which was almost half of the revised EHP estimated required expenditure per capita per annum.

**Discussion:**

The SWAp had invested in some very cost-effective health interventions. In terms of numbers of patients treated, the EHP had delivered two thirds of the services required. This was despite serious under-funding of the EHP, an increase in the population and shortage of staff.

**Conclusions:**

The identification of interventions of proven effectiveness and good value for money and earmarked funding through a SWAp process can produce measurable improvement in health service delivery at extremely low cost.

## Background

Donors may be prepared to relinquish individual health projects and contribute to basket funding through a SWAp so long as the health sector plans for which the basket funding are used address health priorities in an efficient and effective way. An EHP is commonly used in a sector wide approach to identify cost-effective interventions chosen to address the most important health problems of the country [1]. In a world of rational planning the outcomes of these chosen interventions would be measured and the reduction in burden of disease calculated. This would provide a way of assessing the overall cost-effectiveness of the approach. Unfortunately measuring outcomes is not possible in countries like Malawi, which does not have a vital registration system or surveys to measure changes in morbidity. Instead, alternative ways of assessing how well the approach has performed are required. This paper combines two ways to assess the SWAp use of the EHP in Malawi.

The first way assesses whether sufficient services are being provided to meet the essential health needs. It uses estimates of incidences of the common diseases occurring annually in the country. These are derived from work to measure the burden of disease in the country based on a WHO methodology [2,3]. The incidences are compared to the numbers of patients treated for the diseases which are included in the EHP. Services may be overprovided for some diseases and underprovided for others. The analysis provides an estimate of the coverage for the specific interventions included in the EHP. The limitation of the approach is that the analysis does not measure outcomes or the quality of the service provided. But adequate coverage is a prerequisite of an adequate response to the identified health needs of the population and so is a useful first measure of success.

The second way to assess the EHP is to review the choice of interventions contained in the package and identify those interventions that are tackling the most important diseases and are good value for money. Recent published work has identified cost-effective assessments of many interventions in tropical populations [4]. Are there interventions that would be more useful than those in the existing EHP?

A combination of an assessment of the choice and coverage of an intervention in an EHP provides a proximate measure of its usefulness. This assessment combined with knowing the overall cost of the EHP allows SWAp partners to gauge its value and therefore the SWAp's contribution to the health sector.

What have other countries using a SWAp done to assess performance? The SWAp in one country is always different from that in another; some emphasise its role in strengthening the policy framework, some the financial framework, while others the institutional framework of its health sector [5]. Assessments of SWAp have proved problematic whatever its prime purpose [1]. The lack of outcome data is widespread [6]. The structure and content of a SWAp vary between countries [7] and the formal structure of the relationships between donors and recipients does not necessarily withstand policy challenges [8]. A recent review in Zambia found a limited increase in administrative efficiency, a reduction in technical efficiency as shown by a drop in bed utilisation and no improvement in allocative efficiency [9]. Our study was designed to look solely at technical efficiency, which we define as the efficient delivery of health care to a population, through an analysis of the appropriateness of the EHP interventions and their coverage in 2008.

## Methods

### Estimating the burden of disease

WHO provided estimates of the burden of disease of Malawi in 2002 and these were assessed and found to be robust and useful for planning purposes [3]. Since 2002 the occurrence of some diseases has changed in Malawi. Recent population based surveys indicate reductions in mortality. The latest such survey, a multiple indicator cluster survey (MICS 2006) describes reduced child and maternal mortality and an increase in impregnated bed net use [10]. A recent report on HIV prevalence prepared by the Ministry of Health (MOH) estimates the recent trend in HIV prevalence and deaths from AIDS [11], indicating that HIV prevalence has levelled off but deaths have increased. The burden of disease model was adjusted to take into account these recent estimates of morbidity and mortality. Incidence of the leading causes of disease burden as ranked by disability adjusted life years (DALYs) were derived from the model and used to calculate the number of cases needed to be served per 1000 population. Estimates of required coverage used in a recently revised EHP costing exercise were used as default values for interventions such as family planning and immunisations.

### Estimating coverage by the chosen EHP interventions

The health management information system (HMIS) has been collecting performance data since 2001 and offers evidence of changes in use of health facilities. Data collected in the first year were not complete; more recent data are more robust and can be analysed with some degree of confidence [12]. The number of cases of each EHP intervention provided in 2007/8 was calculated per 1000 population.

The rate ratio of provision (number of cases provided for per 1000 population) to need (the number of cases needed to be served per 1000 population) was calculated for each EHP intervention. This gave an estimate of the gap between what was provided and what was required, and provided an assessment of how well the EHP had met its objectives and how much more (or less) there was to do.

### Assessing the cost-effectiveness of current and potential EHP interventions

The value of US$ 150 per DALY was used as the cut-off point for cost-effective interventions. This was based on recommendations of the WHO Ad Hoc Committee on Health Research Priorities, which suggested that any intervention costing less than US$ 150 per DALY averted should be considered attractive in low-income countries [13]. Values were taken from the publication - Disease Control Priorities in Developing Countries [4], except for rapid diagnostic tests for malaria which were taken from a more recent publication [14]. Each intervention already in the EHP was assessed to see if the chosen intervention was cost-effective. All other interventions for which cost-effective studies have been undertaken and which could be potentially useful in reducing the top 20 diseases ranked by contribution to the burden of disease (in DALYS) were also assessed.

### Identifying EHP expenditure

Expenditure figures were derived from National Health Accounts (NHAs) for financial years 2002/03 to 2005/6. NHAs figures were not available for 2006/07 and 2007/08 financial years and as such total donors, public and private expenditures were not available for these years. Instead, government and donor SWAp (pooled and earmarked) actual expenditure data from the MOH were used to demonstrate the level of resources available for the EHP for these financial years. The expenditure figures did not include other funding sources such as households, firms and parastatals, international non-governmental organisations (NGOs) and donors outside the SWAp.

## Results

### Burden of disease estimates

The total burden of disease (7.5 million DALYS) and the top 20 ranked disease groups were estimated for 2008 (Table 1). HIV/AIDS was the top disease followed by lower respiratory infections and malaria.

**Table 1:** Leading causes of burden of disease in 2008 - Malawi

***Rank ***	Persons	***% total DALYs***	Males	***% total DALYs***	Females	***% total DALYs***
1	HIV/AIDS	28.9	HIV/AIDS	24.9	HIV/AIDS	32.7
2	Lower respiratory infections	12.0	Lower respiratory infections	13.1	Lower respiratory infections	10.9
3	Malaria	8.6	Malaria	8.9	Malaria	8.4
4	Diarrhoeal diseases	8.4	Diarrhoeal diseases	8.8	Diarrhoeal diseases	8.0
5	Conditions arising during the perinatal period	4.2	Conditions arising during the perinatal period	5.9	Conditions arising during the perinatal period	2.6
6	Tuberculosis	2.2	Tuberculosis	2.9	Protein-energy malnutrition	1.8
7	Protein-energy malnutrition	1.9	Road traffic accidents	2.2	Tuberculosis	1.5
8	Road traffic accidents	1.6	Protein-energy malnutrition	2.0	Cataracts	1.3
9	Cataracts	1.1	Drownings	1.2	Unipolar depressive disorders	1.2
10	Unipolar depressive disorders	1.0	Cataracts	1.0	Abortion	1.1
11	Cerebrovascular disease	0.8	Violence	0.8	Road traffic accidents	1.0
12	Drownings	0.7	Lymphatic filariasis	0.8	Maternal sepsis	1.0
13	Ischaemic heart disease	0.7	Ischaemic heart disease	0.8	Cerebrovascular disease	0.9
14	Abortion	0.6	Unipolar depressive disorders	0.8	Maternal haemorrhage	0.8
15	Iron-deficiency anaemia	0.5	Cerebrovascular disease	0.7	Trachoma	0.7
16	Iodine deficiency	0.5	Drug use disorders	0.6	Ischaemic heart disease	0.6
17	Congenital anomalies	0.5	Congenital anomalies	0.6	Iron-deficiency anaemia	0.6
18	Asthma	0.5	Fires	0.6	Iodine deficiency	0.5
19	Lymphatic filariasis	0.5	Iodine deficiency	0.6	Asthma	0.5
20	Violence	0.5	Asthma	0.6	Congenital anomalies	0.5

The burden in children under 15 years of age was 2.1 million DALYS, which accounted for 29% of the total burden of disease in Malawi. Pneumonia was the top disease followed by diarrhoeal disease and then malaria. Malnutrition and neonatal illnesses took fifth and six places in rank order after HIV/AIDS.

The burden of disease for adults was 72% of the total burden of disease at 5.4 million DALYs. The leading causes in adults were HIV/AIDS, followed by TB and cataracts. TB and lower respiratory infection, which was fourth place in adults, were likely to have been associated with the HIV epidemic. Disabling conditions - cataract, psychiatric disorders, and filariasis - caused significant burden, being third, fifth and 17^th ^in the rank order. Road traffic accidents were sixth in rank, followed by reproductive health disorders despite affecting only half the population. Indeed women bore a heavier burden of disease than men - 59% in women as compared to 46% in men. Disease burden due to abortion - meaning miscarriage and the effect of induced (criminal) abortion - was the second most important disease in women.

Incidence estimates for each disease were available from the global burden of disease (GBD) model. These were used to calculate the number of patients requiring the EHP interventions.

### Estimating coverage of the EHP interventions

**Figure 1:**
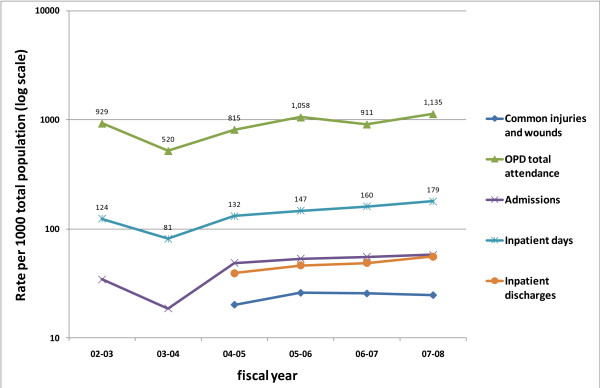
General activity health sector - Malawi 2002 - 2008.

Coverage had increased for most services such as for reproductive health, child services, immunisations and HIV/AIDS since the SWAp started (Table 2). The rate of out patient attendances and inpatient days per 1000 population had both increased from 929 attendances in 2002/03 to 1135 in 2007/08 and from 124 inpatient days in 2002/03 to 179 in 2007/08 (Figure 1). The treatment of acute respiratory infection in under 5 year old children increased from 265/1000 to 348/1000 between 2004/05 and 2007/08.

**Table 2:** Service provision of selected EHP interventions in all health facilities in Malawi from 2002/3 to 2007/8

Data element from HMIS	Denominator per 1000	Financial year					
		02-03	03-04	04-05	05-06	06-07	07-08
Antenatal total visits	live births	3057	1920	2694	2475	2320	2486
Delivery by skilled personnel	live births	422	266	453	416	422	493
Woman with obstetric complication treated at obstetric care facility	live births	46	28	30	31	33	39
Caesarean section	live births	19	11	20	23	22	26
Abortion complications treated	live births			16	17	21	23
Woman of reproductive age receiving modern family planning methods	Female 15-45	651	435	221	159	137	160
BCG	live births	633	561	936	951	957	1042
Pentavalent III	live births	569	465	695	796	808	910
Polio-III	live births	570	464	800	786	803	920
Measles 1st dose at 9 months	live births	492	368	658	691	728	857
Vitamin A dose to 6 - 59 months population	Under 5	323	0	373	372	346	579
Volunteer counselling confidential test and serostatus result 15-49 y	Adult 15+	12	0	26	43	66	103
HIV test positive 15-49 years	Adult 15+			6	10	13	20
HIV positive person receiving anti-retroviral treatment	Adult 15+			2	9	21	18
Pregnant woman receiving VCT and serostatus result	Female 15-45			5	17	31	48
Pregnant woman tested HIV positive	live births			9	25	43	60
Nevirapine dose to baby born to woman with HIV	live births			1	2	2	4
Home-based Care patient followed-up and provided treatment	Adult 15+			8	13	17	23
Case treated as STI - new	Adult 15+			29	27	24	23
Child attending under-five clinic	Under 5	2081	1321	2116	2193	2239	2571
Acute Respiratory Infections under 5 years - new	Under 5			265	337	338	348
Diarrhoea non-bloody under 5 years - new	Under 5	108	64	122	148	119	130
Malnutrition under 5 years - new	Under 5	65	28	34	48	41	32
Malaria under 5 years - new	Under 5			657	849	831	999
Malaria 5 years and older - new	Over 5			194	215	220	251
Dysentery - new	Total	6	4	7	7	8	8
Eye infection - new	Total			26	32	23	27
Ear infection - new	Total		6	10	12	10	10
Skin infection - new	Total			32	40	34	37
Oral condition - new	Total			25	29	27	29
Schistosomiasis - new	Total			5	6	8	7
Leprosy - new	Total			0	0	0	0
Common injuries and wounds	Total			20	26	26	25
OPD total attendance	Total	929	520	815	1058	911	1135
Admissions	Total	35	19	49	53	55	58
Inpatient days	Total	124	81	132	147	160	179
Inpatient discharges	Total			39	46	49	56
Inpatient deaths (excluding maternity)	Total	47	65	38	34	28	30

By 2007/08 the gap between what was required and what was provided was often narrow - the mean gap was 0.68 of estimated need (Table 3). A few services were over-provided, such as the treatment of malaria - probably due to the over-diagnosis of malaria in out-patients. Some services needed to double - such as health facility deliveries, the treatments of acute respiratory infection (ARI) in under 5 year olds, of abortion complications and of acute malnutrition, and supplementary feeding. A few services were even more limited - dealing with complications of pregnancy and the newborn, treatment of diarrhoea and mother to child transmission (MTCT) prevention.

**Table 3:** Gap analysis showing the proportion of health needs met by the Essential Health Package - Malawi 2008

Interventions of the existing Essential Health Package	National Incidence Levels derived from Burden of Disease estimates	Total burden to be met by the EHP as cases per 1000 of the population	Provision in 2007/8 as cases per 1000 of the population	The gap between need and delivery (ratio of provision to need)
		a	b	b/a
Full immunization		58.5	41.9	0.72
Measles		45.1	39.4	0.87
ARI in under-5s	1,829,077	140	68.1	0.49
Malaria - bednets	1,200,000	91.8	91.8	1.00
Malaria - under 5	2,238,248	171.3	195.7	1.14
Malaria - 5 and over	1,932,413	147.9	208.2	1.41
Antenatal Care	797,313	61	45.7	0.75
Normal Delivery	637,850	43.9	22.7	0.52
Delivery complications	115,786	10.4	3.2	0.31
Newborn Complications	127,570	9.8	0.8	0.08
Abortion Complications	55,481	4.2	1.4	0.33
Treatment of Syphilis in Pregnancy	31,095	2.4	2.2	0.92
Postpartum Care	637,850	48.8	13	0.27
Condoms	12,301	6.5	5	0.77
Oral Contraceptive Pill	10,854	7.8	6	0.77
Depo-provera injection	100,582	9.4	7.2	0.77
Norplant	2,894	0.1	0.1	1.00
IUCD	724	0.1	0.1	1.00
Bilateral Tubular Ligation		2.9	2.1	0.72
Vasectomy		0.1	0.1	1.00
Passive Case Detection		6.2	4.4	0.71
Treatment -smear negative and extra-pulmonary TB		3.6	2.6	0.72
Treatment -smear positive TB		0.9	0.6	0.67
Treatment - relapsed cases		0.2	0.2	1.00
Treatment of Dehydration in U5s	3,815,318	292	25.5	0.09
Case management in Cholera		0.1	0.1	1.00
Case management of Dysentery		14.2	8.5	0.60
HIV Testing & Counselling (HTC)	1,191,648	91.2	54.7	0.60
Management of OIs	261,325	20	12	0.60
Screening/treatment of syphilis	148,956	11.4	6.8	0.60
Prevention of MTC transmission	78,137	12.2	0.6	0.05
Testing and Treatment of Other STIs	436,676	33.4	12.8	0.38
CBHBC	37,892	2.9	0.8	0.28
ARV (adult)	79,200	5.6	3.4	0.61
ARV (child)	10,800	0.8	0.5	0.63
ARV Supplementary Feeding (adult)	79,200	1	0.6	0.60
ARV Supplementary Feeding (child)	10,800	0.9	0.5	0.56
Diagnosis and Case Management	4,401,521	11.5	6.9	0.60
Mass Treatment	352,647	27	16.2	0.60
Growth Monitoring of U5 Children	2,383,085	182.4	183.2	1.00
Micronutrient supplementation	2,383,085	182.4	109.4	0.60
Severe Acute Malnutrition (Inpatient)	44,162	3.4	1.4	0.41
Moderate Acute Malnutrition (Outpatient)	40,375	3.1	2.5	0.81
Supplementary Feeding	244,732	18.7	7.7	0.41
Treatment of conjunctivitis		45.6	27.3	0.60
Acute otitis media in under 5s	1,089,317	7.3	4.4	0.60
Scabies	-	62.6	37.6	0.60
Treatment of Fractures and Dislocations	164,244	12.6	10.1	0.80
Treatment of Wounds	213,765	16.4	15.3	0.93
**Total interventions**	** **	**1931.6**	**1311.3**	**0.68**

### The cost-effectiveness of actual and potential EHP interventions

**Figure 2:**
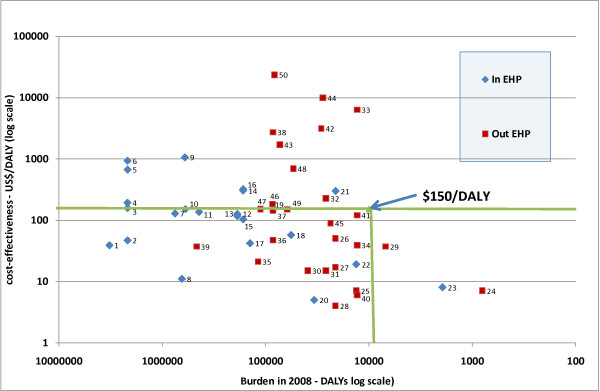
**The cost-effectiveness of and relationship to disease burden of actual and potential EHP interventions - Malawi 2008. **1 IMCI, 2 HIV Testing & Counselling (HTC), 3 Management of OIs, 4 Prevention of MTC transmission, 5 CBHBC, 6 ARV, 7 ARI in under-5s, 8 Malaria - bednets, 9 Treatment of Dehydration in U5s using Tanzi, 10 Malaria - under 5 using ACT, 11 Treatment of Wounds, fractures and dislocations, 12 Maternal care, 13 Family planning, 14 Treatment -smear negative and extra-pulmonary TB, 15 Treatment -smear positive TB, 16 Treatment - relapsed cases, 17 Growth Monitoring of U5 Children, 18 Testing and Treatment of Other Sexually Transmitted Infections (STIs), 19 Malaria - 5 and over using ACT, 20 Schistosomiasis mass treatment, 21 Full immunization with Penta vaccine, 22 Intermittent preventive treatment in pregnancy with SP, 23 Measles, 24 DPT, 25 Intermittent preventive treatment in children with SP, 26 Rapid diagnostic tests to improve malaria treatment, 27 Indoor residual spraying (two rounds per year), 28 Home made ORS, 29 Onchocerciasis, 30 Mass treatment filariasis, 31 Case finding and treatment of Trypanosomiasis, 32 Supplementary Feeding, 33 Mass treatment of Trachoma, 34 Trachoma surgery, 35 Prevention of Road Traffic Accidents, 36 Improved water supply, 37 Improved sanitation, 38 Cholera or rotavirus immunisation, 39 School health, 40 Emergency medical care - first aid training of volunteers, 41 Emergency medical care - ambulance service, 42 Bipolar disorders, 43 Depression, 44 Schizophrenia, 45 Epilepsy, 46 Cataract extraction, 47 ACE inhibitors, b-blockers and diuretics for CCF, 48 Aspirin, b-blockers and ACE inhibitors for IHD, 49 Aspirin for stroke, 50 Cancer

33 of the 55 EHP interventions were found to be potentially cost-effective (<$150/DALY), 12 were not so cost-effective (>$150/DALY) and cost-effective estimates were not available for 10 (Figure 2 and Additional File 1). 15 potential interventions, which were cost-effective and tackling one of the top 20 ranked diseases, were identified.

Pentavaccine was much less cost-effective ($298 per DALY) than the basic DTP and measles vaccines, which cost $7 to avert 1 DALY. Of interventions for TB, only the directly observed short course treatment strategy (DOTS) was cost-effective using the <$150/DALY criterion. The chosen diarrhoeal disease intervention was very expensive at $1060/DALY and the home remedy option would have been much cheaper at $4/DALY. Antiretroviral therapy (ART) was clearly not cost-effective at $922/DALY, along with other treatments of AIDS in comparison with many other interventions. Supplementary feeding for children was not cost-effective at $225/DALY. All the other chosen interventions were cost-effective.

In terms of potential interventions not yet included in the EHP, those dealing with diseases which warranted consideration because of their high burden rank were new forms of malaria control, home made oral rehydration solution, mass treatment of soil helminths and filariasis, control measures for trypanosomiasis, treatment and control of trachoma, prevention of road traffic accidents, district hospital based surgery, treatment of cataracts, mental illness, epilepsy, ischaemic heart disease and stroke. In addition there were interventions such as environmental health improvements, school health, integrated management of childhood illnesses (IMCI) and first aid that would also tackle high burden diseases.

However, not all the potentially new interventions were cost-effective. The ones which were less than $150 per DALY were intermittent prophylactic prevention treatment (IPPT) of malaria in children at $3-12/DALY, rapid diagnostic testing for malaria, indoor residual spraying for malaria control, home made oral rehydration solution (ORS), mass treatment of soil helminths, filariasis, onchocerciasis and trypanosomiasis, prevention of road traffic accidents, problems requiring surgery, improved water supplies and sanitation, IMCI, school health, emergency services (ambulances and first aid) and treatment of epilepsy and aspirin for stroke.

### Overall cost of the EHP

**Figure 3:**
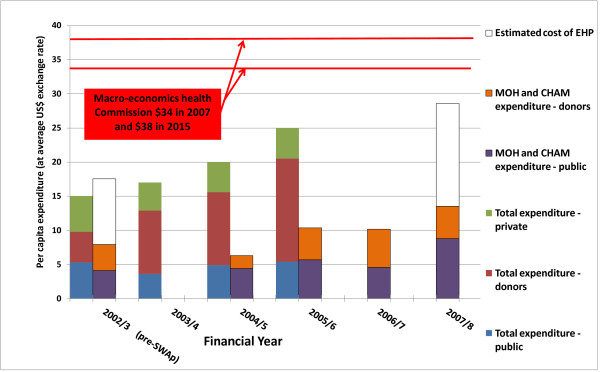
**Cost of Essential Health Package-Malawi**. Per capita national expenditure on health (excluding research, training, environment and nutrition) for 2002/3 to 2005/6 (left hand column of each year), per capita expenditure on EHP by government and SWAp donors for 2002/3 and 2004/5 to 2007/8 (right hand column of each year)

In 2005/06 the national total expenditure on health (excluding research, training, nutrition and environment but including out of pocket expenditure) was $25 per capita (Figure 3), which was significantly short of the Macroeconomics Commission estimated minimum cost of $34 per capita in 2007 rising to $38 per capita in 2015 for essential services [15]. Over the first four years of the SWAp period the funding provided 57% of the necessary minimum financial resources needed. The funding gap between national health expenditure and the $34 per capita target reduced from 44% to 74% between 2002/3 (the financial year before SWAp started) and 2005/6. More recent figures were not available to identify trends of total health expenditure after 2005/06 but expenditure for the EHP provided by SWAp partners rose from $7.9 in 2002/3 to $13.5 per capita in 2007/8. These figures can be compared with the estimated cost of the EHP in 2002/3 of $17.3 per capita and revised in 2007/8 to $28.6 per capita. SWAp funding had increased by 70% between 2002/3 and 2007/8 but by 2007/8 had not reached the expenditure planned for 2002/3.

## Discussion

### Is the choice of EHP interventions appropriate?

Much of the cost-effectiveness analysis has been published since the original choice for the Malawi EHP. For instance the WHO-CHOICE initiative started in 2002 after the EHP had been chosen [16]. The considerable harmony between that choice and the list of interventions which have subsequently been found to be good value can be seen as a considerable success. The exclusions from that list are also notable. There was no cancer care; ART were introduced with the 3 by 5 initiative after the EHP was originally designed; mental illness was not included. The exclusions were tough and appropriate.

The three weak choices were (i) the use of commercial ORS, which was a policy error really belonging to WHO and UNICEF [17], (ii) supplementary feeding of malnourished children - another UNICEF/World Bank sacred cow, which has never been found to be cost-effective [18] and (iii) penta vaccine. If GAVI were to fail to fund this expensive option in the future then the country could revert to the more basic and cost-effective diphtheria, tetanus and pertussis (DTP) vaccine regime. ART deserves special consideration. It is clearly outside the cost-effectiveness bracket of the EHP. Again, like penta vaccine, it is wholly funded by an outside donor and if Malawi had asked for Global funds to be used for other interventions outside its remit, it is certain that the Global Fund would have rejected the request. In terms of cost-effectiveness of the EHP, so long as the Global fund continues to fund ART and SWAp resources are not diverted from more valuable interventions, the integrity of the choice of the EHP can be maintained. The issue of staff being diverted from the EHP by ART clinics is well documented and needs to be tackled [19].

There are some exciting interventions that can be considered for inclusion in a revised EHP. The decision to include them depends on whether donors and the Government of Malawi (GOM) are prepared to fully fund the current EHP. Fully funding the existing EHP before embarking on new interventions is probably an important principle to follow. However, the list is tempting because the interventions are really good value for money. Their cost effectiveness ratios are all below $150/DALY, considerably less than the thresholds (measured in $/DALY) used in more developed countries such as India ($2500), China ($5000), South Africa ($15,000) or England ($50,000) [20].

### Is coverage adequate?

Despite the increase in the population, from 9.9 million in 1998 to 13.1 million in 2008 [21], and the AIDS epidemic and despite the planned under-funding of the original EHP, the gap between what was needed and what was provided was not wide. In terms of numbers of patients treated, the EHP had delivered two thirds of the services required. This can be considered a success. Another study conducted by the Ministry of Health found the equitable distribution of services had improved over the last decade [22] suggesting that the quality as well as the quantity of coverage had improved. The EHP is the chosen vehicle for the health sector's contribution to poverty reduction, and this provides some evidence of its achievement in reducing inequalities. However, quality of care is likely to have been compromised. The lack of staff and the huge numbers of patients make this almost inevitable [19]. As new staff from the emergency human resources plan fill vacancies, improvements in quality will be possible, a pre-requisite to reducing the burden of disease.

The assessment of coverage was dependent on HMIS data and therefore any analysis was limited by the data quality. As identified in a recent Health Metrics report on the health information systems in Malawi while HMIS was present in all health facilities it was deemed inadequate, with capacity and practices as well as dissemination needing improvements [12]. However the coverage estimates are probably accurate enough for their use at national level for this type of analysis where annual totals of common interventions were used.

### Is funding adequate?

The EHP expenditure figures only included SWAp and government funds. This is not a major drawback since the EHP was designed and costed by government as a tool for priority setting and to be funded by both government and partners through a SWAp. The other sources of funds such as households, international NGOs, private firms and parastatals and donors outside the SWAp were not party to this EHP and its funding arrangements and indeed the MOH and partners in the SWAp have no control on what they spend their funds. Their control is on resources in the SWAp (pooled and earmarked) which are meant to be spent on EHP.

Funding at the start of the SWAp was approximately half the required amount - $18.4 as compared to $34 a head - the base estimate from the Macroeconomics and Health Commission. The revised costing of the EHP estimated resources required to be $28.6 per capita, with the increase largely coming from the Global Fund money to pay for ART and associated AIDS care, new artemesinin combination therapy and a maternity "Road Map" to increase deliveries attended by skilled midwives. The actual disbursement had always been less than pledges from donors between 2002/3 and 2007/8, when in this period only 62% of pledges were disbursed [23]. The net result was a serious under-funding of almost half of the revised EHP estimated required expenditure per capita per annum.

### Technical efficiency

We were not able to measure outcomes. Taking antibiotic treatment of children with pneumonia as an example, it would have been desirable to measure changes in mortality in under 5 year old children from pneumonia. Instead we have confirmed a number of things. First, the identification and appropriate treatment of lower respiratory infections though child clinics ($129 per DALY saved) and IMCI ($39 per DALY saved) is potentially a cost-effective intervention. Second, the disease causes the biggest disease burden in Malawi children. Third, the EHP delivered 68.1 treatments per 1000 in a population with a need for 140 treatments per 1000, i.e. half the required service. This was despite the increase in the treatment rate of acute respiratory infections from 265 per 1000 under 5 children in 2004/5 to 348/1000 in 2007/8. For this particular intervention the gap between need and provision remained wide.

A serious limitation of our study was the lack of information about the quality of the appropriate intervention - were children with pneumonia getting the correct diagnosis and treatment? Operations research of IMCI in Malawi has found significant numbers of missed diagnoses and incorrect treatment.

A similar assessment was made for each of the EHP interventions to assess these components of technical efficiency. Quality assessment was available for some, such as for caesarean section for disproportion [24]. However, the study found that the data to assess the quality of care were limited and hence our conclusions can only relate to coverage and appropriateness of the EHP interventions.

## Conclusions

This assessment has shown that SWAp had invested in some very cost-effective health interventions - indeed a portfolio of interventions of far better value for money than the range offered by donor governments in their own countries. Despite serious under-funding of the EHP, Malawi had achieved measurable improvements in service delivery. This had been achieved in the face of a human resource crisis, partly caused by a brain drain of health staff and an AIDS epidemic. It is concluded that the identification of interventions of proven effectiveness and earmarked funding through a SWAp process can produce measurable improvements in health service delivery at extremely low cost.

## Competing interests

The authors declare that they have no competing interests.

## Authors' contributions

CB undertook the analysis of the burden of disease, the gap analysis and the assessment of cost-effectiveness. TM undertook the financial analysis. CB wrote the first draft of the manuscript. Both were responsible for revisions and both authors read and approved the final manuscript.

## Supplementary Material

Additional file 1 **Cost-effectiveness and importance of actual and potential interventions - Malawi 2008. **Cost-effective ratios (US$/DALY) of existing and potential EHP interventions, categorised by (i) being under or over $150/DALY threshold, (ii) the intervention dealing with one of the top 20 diseases, (iii) an intervention found to be both cost effective and high ranking for Malawi in 2008. Click here for file
