# Supplementary material for: Assessing the use of an essential health package in a sector wide approach in Malawi
Source: Health Res Policy Syst. 2011 Jan 17;9:4. doi: 10.1186/1478-4505-9-4 (PMC3032754; doi:10.1186/1478-4505-9-4)
Supplement: Additional file 1 — Cost-effectiveness and importance of actual and potential interventions - Malawi 2008. Cost-effective ratios (US$/DALY) of existing and potential EHP interventions, categorised by (i) being under or over $150/DALY threshold, (ii) the intervention dealing with one of the top 20 diseases, (iii) an intervention found to be both cost effective and high ranking for Malawi in 2008. [file 1478-4505-9-4-S1.DOC]

### Additional file 1 – Cost-effectiveness and importance of actual and potential interventions – Malawi 2008

| **EHP Component** | **Intervention** | **Inter-vention Number** | **cost-effectiveness - US$/DALY average and range** | | | **EHP inter-ventions >150$/DALY** | **Top 20 DOB conditions** | **Potential new interventions - top 20 and <150$/DALY** |
| --- | --- | --- | --- | --- | --- | --- | --- | --- |
|  |  |  | **Mean** | **Low** | **High** |  |  |  |
| **Vaccine-preventable** | | |  |  |  |  |  |  |
|  | Full immunization with Penta vaccine | 1.1 | 298 | 7 for DTP | 8 | >150$/DALY | Prevented |  |
|  | Measles | 1.2 |  | 8 |  |  | Prevented |  |
| **ARI** | | |  |  |  |  |  |  |
|  | ARI in under-5s | 2.1 | 129 | 50 | 208 |  | Yes |  |
| **Malaria** | | |  |  |  |  |  |  |
|  | Malaria - bednets | 3.1 | 11 | 5 | 17 |  | Yes |  |
|  | Malaria - under 5 using ACT | 3.2 |  | ? | 150 |  | Yes |  |
|  | Malaria - 5 and over using ACT | 3.3 |  | ? | 150 |  | Yes |  |
|  | Intermittent preventive treatment in pregnancy with SP | 4.1 | 19 | 13 | 24 |  | Yes |  |
|  | Intermittent preventive treatment in children with SP | Potential |  | 3 | 12 |  | Yes | Yes |
|  | Rapid diagnostic tests to improve malaria treatment | Potential |  | ? | 75 |  | Yes | Yes |
|  | Indoor residual spraying (two rounds per year) | Potential | 17 | 9 | 24 |  | Yes | Yes |
| **Adverse Maternal/Neonatal Outcomes** | | |  |  |  |  |  |  |
|  | Antenatal Care | 4.1 | 127 | 82 | 409 |  | Yes |  |
|  | Normal Delivery | 4.2 |  |  |  |  | Yes |  |
|  | Postpartum Haemorrhage | 4.3 |  |  |  |  | Yes |  |
|  | Eclampsia | 4.4 |  |  |  |  | Yes |  |
|  | Obstructed Labour | 4.5 |  |  |  |  | Yes |  |
|  | Severe Anaemia | 4.6 |  |  |  |  | Yes |  |
|  | Sepsis | 4.7 |  |  |  |  | Yes |  |
|  | Newborn Complications | 4.8 |  |  |  |  | Yes |  |
|  | Abortion Complications | 4.9 |  |  |  |  | Yes |  |
|  | Treatment of Syphillis in Pregnancy | 4.10 |  |  |  |  | Yes |  |
|  | Postpartum Care | 4.11 |  |  |  |  | Yes |  |
|  | Condoms | 4.12.1 | 117 |  |  |  |  |  |
|  | Oral Contraceptive Pill | 4.12.2 |  |  |  |  |  |  |
|  | Depo-provera injection | 4.12.3 |  |  |  |  |  |  |
|  | Norplant | 4.12.4 |  |  |  |  |  |  |
|  | IUCD | 4.12.5 |  |  |  |  |  |  |
|  | Bilateral Tubular Ligation | 4.12.6 |  |  |  |  |  |  |
|  | Vasectomy | 4.12.7 |  |  |  |  |  |  |
| **Tuberculosis** | | |  |  |  |  |  |  |
|  | Passive Case Detection | 5.1 |  | part of 5.3 | |  | Yes |  |
|  | Treatment -smear negative and extra-pulmonary TB | 5.2 | 301 | 84 | 551 | >150$/DALY | Yes |  |
|  | Treatment -smear positive TB | 5.3 | 102 | 15 | 189 |  | Yes |  |
|  | Treatment - relapsed cases | 5.4 | 318 | 208 | 429 | >150$/DALY | Yes |  |
| **Acute Diarrhoeal Diseases** | | |  |  |  |  |  |  |
|  | Treatment of Dehydration in U5s using Tanzi | 6.1 | 1060 | 500 | 1658 | >150$/DALY | Yes |  |
|  | Case management in Cholera | 6.2 | ? | ? | ? |  | No |  |
|  | Case management of Dysentery | 6.3 | ? | ? | ? |  | No |  |
|  | Home made ORS | Potential | 4 | 4 |  |  | Yes | Yes |
|  |  |  |  |  |  |  |  |  |
| **STDs including HIV/AIDS** | | |  |  |  |  |  |  |
|  | HIV Testing & Counselling (HTC) | 7.1 | 47 | 10 | 85 |  | Yes |  |
|  | Management of OIs | 7.2 | 156 | 3 | 310 | >150$/DALY | Yes |  |
|  | Screening/treatment of syphilis | 7.3 | ? | ? | ? |  | Yes |  |
|  | Prevention of MTC transmission | 7.4 | 192 | 7 | 377 | >150$/DALY | Yes |  |
|  | Testing and Treatment of Other Sexually Transmitted Infections (STIs) | 7.5 | 57 | 9 | 105 |  | Yes |  |
|  | CBHBC | 7.6 | 673 |  |  | >150$/DALY | Yes |  |
|  | ARV (adult) | 7.7 | 922 | 350 | 1494 | >150$/DALY | Yes |  |
|  | ARV (child) | 7.8 |  |  |  | >150$/DALY | Yes |  |
|  | ARV Supplementary Feeding (adult) | 7.9 | ? | ? | ? | >150$/DALY | Yes |  |
|  | ARV Supplementary Feeding (child) | 7.1 | ? | ? | ? | >150$/DALY | Yes |  |
| **Schistosomiasis** | | |  |  |  |  |  |  |
|  | Diagnosis and Case Management | 8.1 | ? | ? | ? |  | Yes |  |
|  | Mass Treatment | 8.2 | ? | 3 | 7 |  | Yes |  |
|  | Environmental Management | 8.3 | ? | ? | ? |  | Yes |  |
|  | School based mass treatment combined with soil helminths | Potential | ? | 8 | 19 |  | Yes | Yes |
|  | Onchocerciasis | Potential | 37 |  |  |  | Yes | Yes |
|  | Mass treatment filariasis | Potential | 15 | 4 | 27 |  | Yes | Yes |
|  | Case finding and treatment of Trypanosomiasis | Potential | 15 |  |  |  | Yes | Yes |
| **Nutritional Deficiencies** | | |  |  |  |  |  |  |
|  | Growth Monitoring of U5 Children | 9.1 | 42 |  |  |  | Yes |  |
|  | Micronutrient supplementation | 9.2 |  | 6 | 11 |  | Yes |  |
|  | Severe Acute Malnutrition (Inpatient) | 9.3 | ? | ? | ? |  | Yes |  |
|  | Moderate Acute Malnutrition (Outpatient) | 9.4 | ? | ? | ? |  | Yes |  |
|  | Supplementary Feeding | 9.5 | 225 |  |  | >150$/DALY | Yes |  |
| **Eye, Ear and Skin Conditions** | | |  |  |  |  |  |  |
|  | Treatment of conjunctivitis | 10.1 | ? | ? | ? |  | No |  |
|  | Acute otitis media in under 5s | 10.2 | ? | ? | ? |  | No |  |
|  | Scabies | 10.3 | ? | ? | ? |  | No |  |
|  | Mass treatment of Trachoma | Potential | 6300 |  |  |  | No | No |
|  | Trachoma surgery | Potential | 39 |  |  |  | No | No |
| **Common Injuries and Poisoning** | | |  |  |  |  |  |  |
|  | Treatment of Fractures and Dislocations | 11.1 | 136 | 54 | 217 |  | Yes |  |
|  | Treatment of Wounds | 11.2 |  |  |  |  | Yes |  |
|  | Prevention of Road Traffic Accidents | Potential | 21 | 3 | 38 |  | Yes | Yes |
|  | Problems requiring surgery | Potential | 136 | 54 | 217 |  | Yes | Yes |
|  |  |  |  |  |  |  |  |  |
| **Environmental health** | | |  |  |  |  |  |  |
|  | Improved water supply | Potential | 47 |  |  |  |  | Yes |
|  | Improved sanitation | Potential | 141 | 11 | 270 |  |  | Yes |
|  | Cholera or rotavirus immunisation | Potential | 2712 | 2478 | 2945 |  |  | No |
|  |  |  |  |  |  |  |  |  |
| **Sector health promotion** | | |  |  |  |  |  |  |
|  | IMCI | Potential | 39 |  |  |  |  | Yes |
|  | School health | Potential | 37 |  |  |  |  | Yes |
|  | Emergency medical care - first aid training of volunteers | Potential | 6 |  |  |  |  | Yes |
|  | Emergency medical care - ambulance service | Potential | 120 |  |  |  |  | Yes |
|  |  |  |  |  |  |  |  |  |
| **Other personal interventions** | | |  |  |  |  |  |  |
|  | Bipolar disorders | Potential | 3113 | 2498 | 3728 |  | Yes | No |
|  | Depression | Potential | 1699 | 657 | 2741 |  | Yes | No |
|  | Schizophrenia | Potential | 9834 | 2472 | 17197 |  | Yes | No |
|  | Epilepsy | Potential | 89 |  |  |  | Yes | Yes |
|  | Cataract extraction | Potential | 183 |  |  |  | Yes | No |
|  | ACE inhibitors, b-blockers and diuretics for congestive heart failure | Potential | 150 | 27 | 274 |  | No | No |
|  | Aspirin, b-blockers and ACE inhibitors for ischaemic heart disease | Potential | 688 | 451 | 926 |  | Yes | No |
|  | Aspirin for stroke | Potential | 149 |  |  |  | Yes | Yes |
